# PGCA: An algorithm to link protein groups created from MS/MS data

**DOI:** 10.1371/journal.pone.0177569

**Published:** 2017-05-31

**Authors:** David Kepplinger, Mandeep Takhar, Mayu Sasaki, Zsuzsanna Hollander, Derek Smith, Bruce McManus, W. Robert McMaster, Raymond T. Ng, Gabriela V. Cohen Freue

**Affiliations:** 1 Department of Statistics, University of British Columbia, Vancouver, British Columbia, Canada; 2 NCE CECR PROOF Centre of Excellence, Vancouver, British Columbia, Canada; 3 University of Victoria - Genome BC Proteomics Centre, Victoria, British Columbia, Canada; 4 Department of Medical Genetics, University of British Columbia, Vancouver, British Columbia, Canada; 5 Department of Computer Science, University of British Columbia, Vancouver, British Columbia, Canada; University of Glasgow, UNITED KINGDOM

## Abstract

The quantitation of proteins using shotgun proteomics has gained popularity in the last decades, simplifying sample handling procedures, removing extensive protein separation steps and achieving a relatively high throughput readout. The process starts with the digestion of the protein mixture into peptides, which are then separated by liquid chromatography and sequenced by tandem mass spectrometry (MS/MS). At the end of the workflow, recovering the identity of the proteins originally present in the sample is often a difficult and ambiguous process, because more than one protein identifier may match a set of peptides identified from the MS/MS spectra. To address this identification problem, many MS/MS data processing software tools combine all plausible protein identifiers matching a common set of peptides into a protein group. However, this solution introduces new challenges in studies with multiple experimental runs, which can be characterized by three main factors: *i)* protein groups’ identifiers are local, i.e., they vary run to run, *ii)* the composition of each group may change across runs, and *iii)* the supporting evidence of proteins within each group may also change across runs. Since in general there is no conclusive evidence about the absence of proteins in the groups, protein groups need to be linked across different runs in subsequent statistical analyses. We propose an algorithm, called Protein Group Code Algorithm (PGCA), to link groups from multiple experimental runs by forming global protein groups from connected local groups. The algorithm is computationally inexpensive and enables the connection and analysis of lists of protein groups across runs needed in biomarkers studies. We illustrate the identification problem and the stability of the PGCA mapping using 65 iTRAQ experimental runs. Further, we use two biomarker studies to show how PGCA enables the discovery of relevant candidate protein group markers with similar but non-identical compositions in different runs.

## Introduction

Shotgun proteomic techniques have gained popularity in the last decades allowing a global and relatively high throughput identification and quantitation of proteins in complex samples such as plasma or total cell/tissue extracts [1, 2]. The appealing peptide-centric nature of shotgun proteomics simplifies sample preparation procedures and does not require extensive protein separation steps as in previous technologies [3]. In a typical experimental workflow the protein mixture is first digested into peptides, which are then separated by liquid chromatography and sequenced by tandem mass spectrometry (MS/MS). The resulting peptide’s fragmentation mass spectra is then used to recover the identity of the proteins originally present in the sample by searching against a sequence database with available software. This process can be decomposed into two main steps. First, the set of peptides resulting from proteolytic digestion of proteins needs to be identified from the MS/MS fragmentation data. Many algorithms have been proposed to match the observed spectra to peptide sequences, including SEQUEST [4], MASCOT [5], X!TANDEM [6], and hEIDI [7] (a comprehensive review of these and other methods can be found in the literature [8–10]). Second, the set of proteins present in the sample before digestion needs to be recovered from the list of identified peptides. Since the same peptide sequences can be present in multiple proteins, the link between proteins originally present in the sample and proteins identified from observed peptides is often lost. Overall, the process of recovering the identities of the original proteins from the observed MS/MS spectra is complex and in most cases ambiguous, adding an important challenge to the analysis and interpretation of the data [3, 8, 11].

Many software tools have been developed to process MS/MS data addressing the protein identification problem, such as ProteinPilot^™^ [12], ProteinProphet [13], Scaffold [14], ProteinLP [10], ProteinLASSO [9], TRIC [15], DBParser [16], and X!TandemPipeline [17]. Although the organization of the results varies among these tools, most of them combine the set of all plausible protein identifiers matching a common set of peptides into a protein group. In accordance with the Molecular & Cellular Proteomics publication guidelines [18], this strategy results in a minimal list of non-redundant protein groups sufficient to explain all observed peptides.

**Fig 1:**
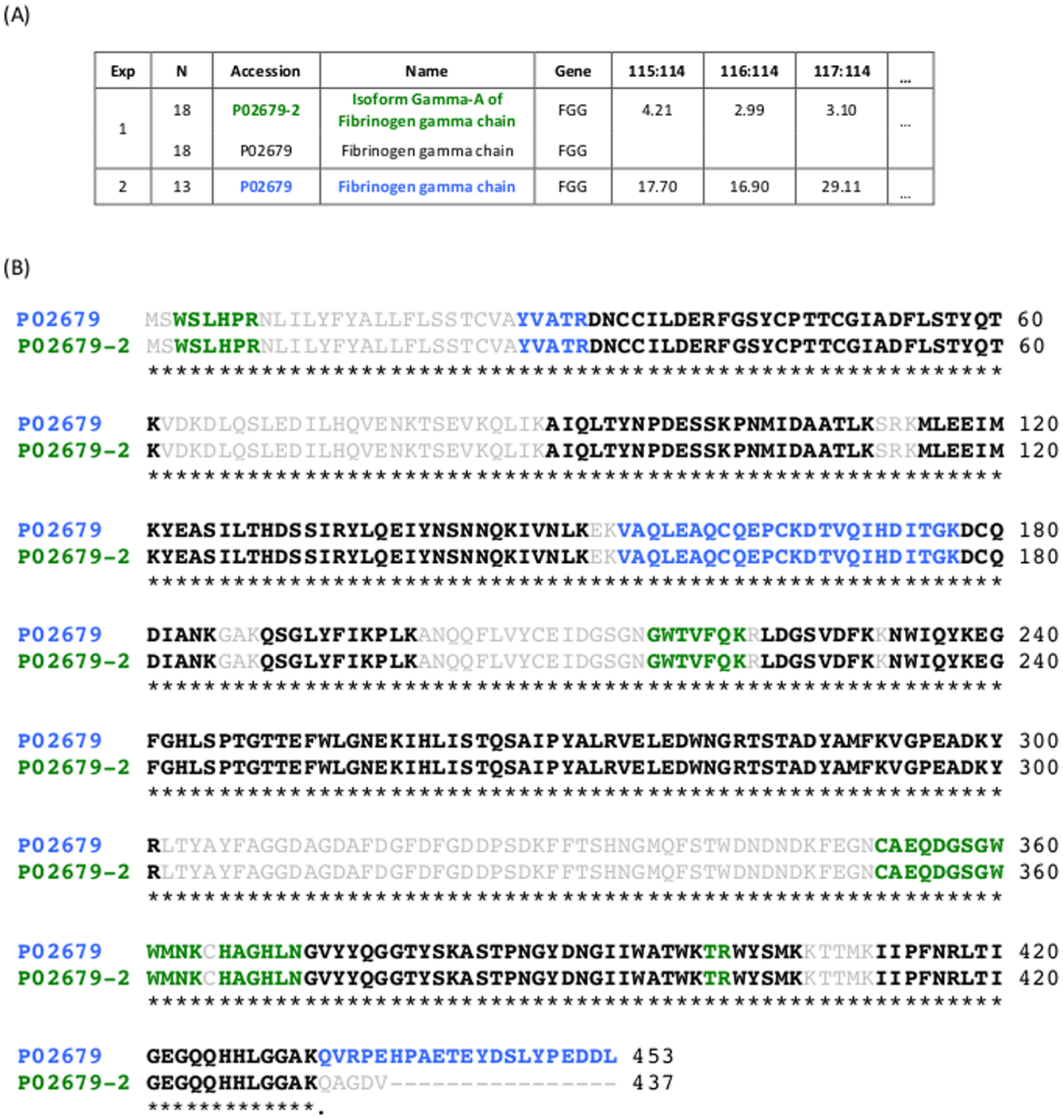
Exemplary challenge in protein identification. (A) A glance at a protein group identified by ProteinPilot in 2 iTRAQ experiments (Exp). Top proteins within each group are shown in bold colour fonts (green fonts for experiment 1 and blue for experiment 2). (B) Coverage maps of the identified protein sequences (aligned with Clustal Omega v1.2.2) with the list of peptides used for identification in bold font. Peptides identified in both experiments are highlighted using black fonts. Peptides identified only in experiment 1 are highlighted in green, and those identified only in experiment 2 are identified in blue fonts.

Fig 1 illustrates the challenge of identifying proteins from the lists of observed peptides with an example from two experimental runs from iTRAQ (isobaric tags for relative and absolute quantitation). For both runs, the raw MS/MS data was processed using ProteinPilot^™^ software v4.0 with the integrated Paragon^™^ Search and ProGroup^™^ algorithms [18] and searching against UniProtKB database (October 2014 release). Fig 1A shows part of the protein summary files for one protein group identified differently in 2 experimental runs (Exp). Within each experiment, ProteinPilot organizes the set of proteins matching the identified peptide list into a group (N represents the local group identifier). Fig 1B shows the aligned amino acid sequences of the proteins identified in these experiments and highlights the peptides identified within each run using different colors and bold fonts (black = identified in both experiments; green = identified only in experiment 1; blue = identified only in experiment 2).

Protein groups with more than one identifier contain, in general, proteins with a high degree of sequence similarity, including multiple entries for one gene product, and isoforms or multiple members of a protein family. Some tools assign a probability or a score to each protein in the group estimating the likelihood that the corresponding protein was present in the sample (see [3, 8] for a review). In most cases, a “winner” (or top-) protein identity is selected to represent each group and it is placed at the top in the protein summary file. For example, in the first experiment of Fig 1, both Isoform Gamma-A of Fibrinogen gamma chain (P02679-2) and Fibrinogen gamma chain (P02679) are returned as a group of proteins based on the set of identified peptides (bold black and green in Fig 1B), and Isoform Gamma-A of Fibrinogen gamma chain is selected as the top-protein to represent the group with all quantitative values assigned to it (Exp 1, Fig 1A). In the second experiment, the detection of the peptide QVRPEHPAETEYDSLYPEDDL belonging only to Fibrinogen gamma chain (in blue at the end of P02679 sequence in Fig 1B) resulted in the unique identification of this protein identification in the group (Exp 2, Fig 1A).

In a typical proteomics workflow, the MS/MS data from each experimental run is processed one at a time to identify and quantify the proteins present in each sample or set of samples included in the run. In many studies, multiple runs are required to analyze all the samples. The previous example depicts the challenge of comparing protein levels among different experiments when protein groups, instead of unique protein identifiers, are used to report protein abundances. The most naïve approach to compare protein groups from different experiments is to rely on the top-protein selected by the processing software within each run. Although in some groups the presence of the top-protein is supported by the detection of unique peptides belonging to it, there are many groups containing indistinguishable protein identifications since all the identified peptides belong to the overlapping regions of the protein sequences [3]. In the example shown in Fig 1, both proteins in experiment 1 could have been present in the original sample but using only the top-protein to link this group across experiments will treat Fibrinogen gamma chain (P02679) as non-detected in this experiment.

In general, the identification problem in a multi-experiment setting can be characterized by three main factors: *i)* each protein group has only a local identifier per experimental run (i.e., protein groups identifiers vary run to run), *ii)* the composition of each group may change across runs, and *iii)* the supporting evidence of proteins within each group may also change across runs. For example, the local protein groups in Fig 1 have different IDs in each run (N) and contain two proteins in experiment 1 versus one protein in experiment 2. Still, these two groups are highly related and can be compared quantitatively in a subsequent analysis. While an independent technical validation or a manual inspection of the groups is required to determine which protein(s) from each group was(were) originally present in the sample, such validation will be rarely done on the full lists of groups [8]. Thus, an alternative solution to select and link the information contained in groups from multiple experiments is required to enable a subsequent statistical analysis.

In this paper, we propose a computationally inexpensive algorithm, called Protein Group Code Algorithm (PGCA), to merge protein summaries from multiple experimental quantitative proteomics data. The goal of PGCA is to enable the analysis of quantitative data available for protein groups instead of unique protein identifiers. Although this strategy does not directly address the protein inference problem that resulted in the identification of protein groups, it enables the user to maintain the information available in the groups for subsequent analyses. PGCA creates a mapping (or dictionary) of local into global protein groups and assigns a protein group code (PGC) to each global group in the resulting list. The global PGC can then be used to link lists of protein groups across different experimental runs. We use three datasets to characterize the problem, to examine in detail the mapping created by PGCA, as well as to carefully evaluate the impact of its application in two biomarker studies.

## Results and discussion

In this section we describe the algorithm we developed to merge proteomic data files containing protein groups that resulted from processing MS/MS data. We then use the algorithm to characterize the composition of typical protein summaries containing groups of protein identifications and evaluate the reproducibility of the groups among multiple experimental runs. Further, we illustrate how the protein inference problem may impact on a subsequent statistical analysis. Finally, we demonstrate two useful properties of PGCA: the proposed method is order invariant (i.e., the results do not depend on the order that files are processed) and incremental (i.e., new files can be added to existing dictionaries). Technical details about the datasets used and the algorithm are presented Materials and methods.

### PGCA: The protein group code algorithm

PGCA can be decomposed into three algorithms: **CreateLocalGroups**, **UpdateDictionary**, and **CodingProteinGroups**.

**CreateLocalGroups** algorithm creates a set of *local* or intra-experimental protein groups (LocalGroupSet) from the protein groups available within each input file. Examples of local protein groups include the top-protein of a group, the set of proteins matching to the top gene, or the whole protein group. We can think of this step as a potential refinement of the groups created from MS/MS data to determine the amount of information contained in each group that will be kept for comparisons across multiple runs.

**Fig 2:**
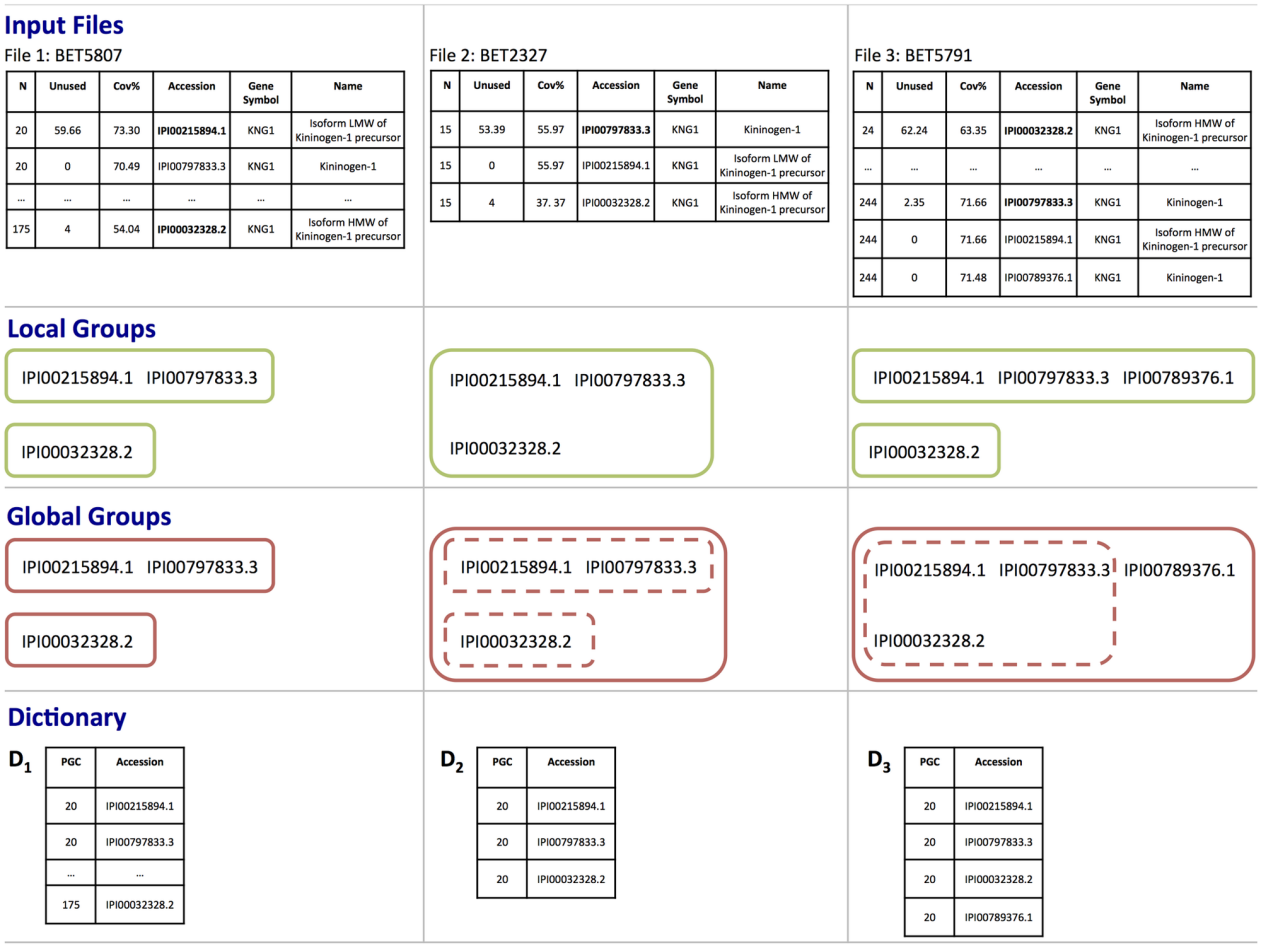
Protein group example. The top panel shows parts of three protein summaries from dataset B that are processed in consecutive order (input files) to construct a dictionary. Bold fonts are used to highlight the top protein in each group as reported by ProteinPilot. The second panel shows the local groups created by CreateLocalGroups algorithm within each file. The third panel shows the global groups created by the UpdateDictionary algorithm. Dotted lines are used to show the global groups of the previous step. The last panel shows the corresponding dictionaries, created by the UpdateDictionary algorithm, as files are being processed. The alignment of IPI00215894.1 (UniProt P01042-2), IPI00797833.3 (almost identical to UniProt P01042-2), and IPI00032328.2 (UniProt P01042-1) are given in S1 Fig.

Fig 2 shows an example of local groups created from three input files. In this example, the local groups are identical to those created by ProteinPilot. The first two files illustrate how the same 3 protein identifiers are organized differently in these two files (i.e., 2 local groups in the first file and 1 local group in the second one). The last file shows how new protein identifiers may be added to existing groups.

**UpdateDictionary** merges sets of local protein groups from different experimental runs creating a set of *global* or inter-experimental protein groups (GlobalGroupSet). Each global protein group is built from the union of connected local groups across multiple runs. A protein group code (PGC) is then assigned to each global group creating a mapping or dictionary.

Fig 2 shows the global group created from the three local groups in the previous example. In the first step, there are two global groups, identical to the local ones, one with 2 proteins and the other one with a single protein. Since the 3 proteins in these global groups appear as a single group in the local group from the second file, PGCA forms a unique global group after processing the second file, with a unique protein group code in the updated dictionary *D*_2_. In the third step, a new protein is added to one of the local groups, and thus to the existing global group. The last panel shows the corresponding dictionaries, created by the UpdateDictionary algorithm, as files are being processed.

**CodingProteinGroups** uses the created dictionary to translate protein identities into protein group codes (PGC) within each protein summary. The assigned PGCs can be used to link the groups across different experimental runs and analyze the quantitative value of each group across experiments.

Continuing with the previous example, after processing all 3 files, a new column is added to each file in the top panel with the resulting PGC, e.g., PGC 20 in this example.

PGCA offers three different approaches enabling the user to tune the degree of similarity among linked protein groups: *i)* **PGCA-All** links the raw protein groups identified within each experimental run, *ii)* **PGCA-topGene** links subsets of proteins matching to the gene of the top protein, and *iii)* **PGCA-topIdentifier** links groups by their top-proteins across different experimental runs. This last option was included in the algorithm for completion. However, we note that there are different ways of doing this without **PGCA** and that the **UpdateDictionary** step becomes trivial in this case since all local groups are singletons.

**Fig 3:**
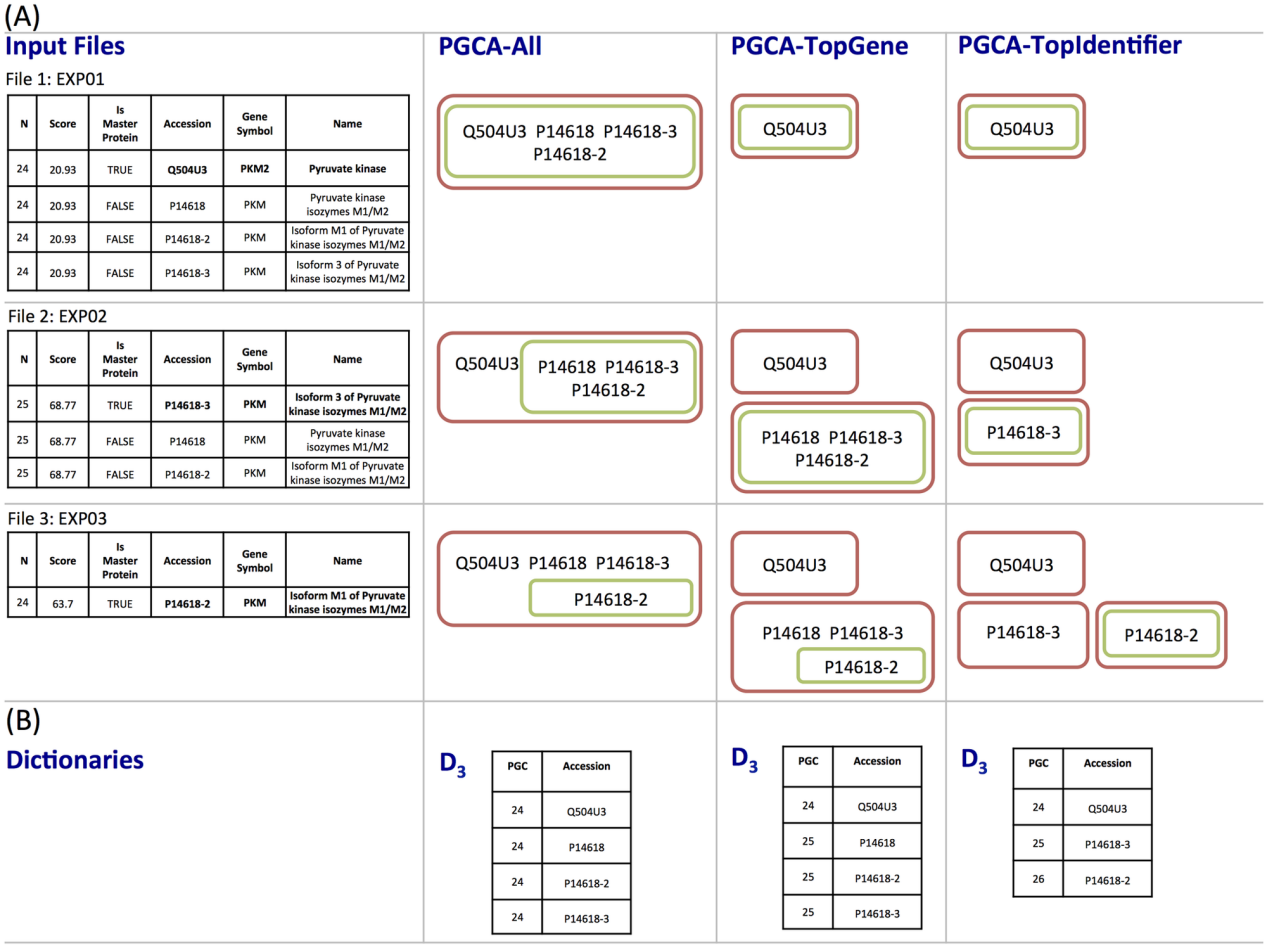
PGCA-All, PGCA-topGene, and PGCA-topIdentifier. (A) The first column shows parts of three protein summaries from dataset C that are processed in consecutive order (input files) to construct a dictionary (*D*_3_) using different PGCA approaches. Bold fonts are used to highlight the top protein in each group as reported by Proteome Discoverer. The next 3 columns show the local groups (green sets) and the global groups (red sets) created by PGCA-All, PGCA-topGene, and PGCA-topIdentifier, respectively, as files are being processed. (B) Dictionaries created by each approach after processing the three input files. The alignment of the four proteins are given in S2 Fig.

Fig 3 illustrates the difference between these approaches using 3 input files. The local groups created by PGCA-All contain all identified proteins within each file. Since none of the proteins in the first file (EXP01) match to the top-gene (PKM2), the local group created by PGCA-topGenes for this file contains only the top protein (Q504U3). In the other two files, all proteins match to the same gene (PKM), thus the local groups created by PGCA-All and PGCA-topGenes are identical. The local groups of PGCA-topIdentifier retain only the top protein within each group. After processing all three files, PGCA-All creates a unique global group with all related proteins; PGCA-topGene creates two global groups since proteins in those groups do not match the same gene; and PGCA-topIdentifier creates three distinct groups since a different master protein was assigned by Proteome Discoverer within each file.

Although some of the groups change their composition and/or ordering of the proteins, in general they contain some common proteins that allow the connection of the detected groups through all runs. PGCA-All assigns a unique and common protein group code (PGC) to all connected proteins, which can then be used to link and analyze this group across runs. Noteworthy, the statistical analysis of some groups can be lost if only the top-identifier is used to link the identified groups across runs. In the example illustrated in Fig 3, different proteins are chosen to represent the group within each run, thus this protein group can not be compared among samples. Different criteria on linking groups from multiple runs can have an important impact in a subsequence statistical analysis.

### Variation in protein identification

Dataset A consists of 4 lists of protein groups generated by ProteinPilot from 4 iTRAQ experiments used to process technical replicates of a pool of plasma from healthy individuals (see Materials and methods and S1 File). Ideally, the 4 lists of protein identifications from each of these experiments should be identical since technical replicates of the same sample were processed in all runs. However, in practice this is not the case due to different sources of variation, including sample preparation, separation, and mass spectrometer detection. On average, 193 protein groups were identified per experimental run, with almost half of them containing a single protein identifier and less than 12% containing proteins matching to multiple genes. Out of the 175 identified groups in the first run, only 97 groups contained exactly the same proteins in all the other experiments, with 63 always containing a single identifier (size 1). Out of the 34 non-singleton groups containing the same proteins in all runs, 20 had the same top-protein. Similar results were observed from other runs.

**Fig 4:**
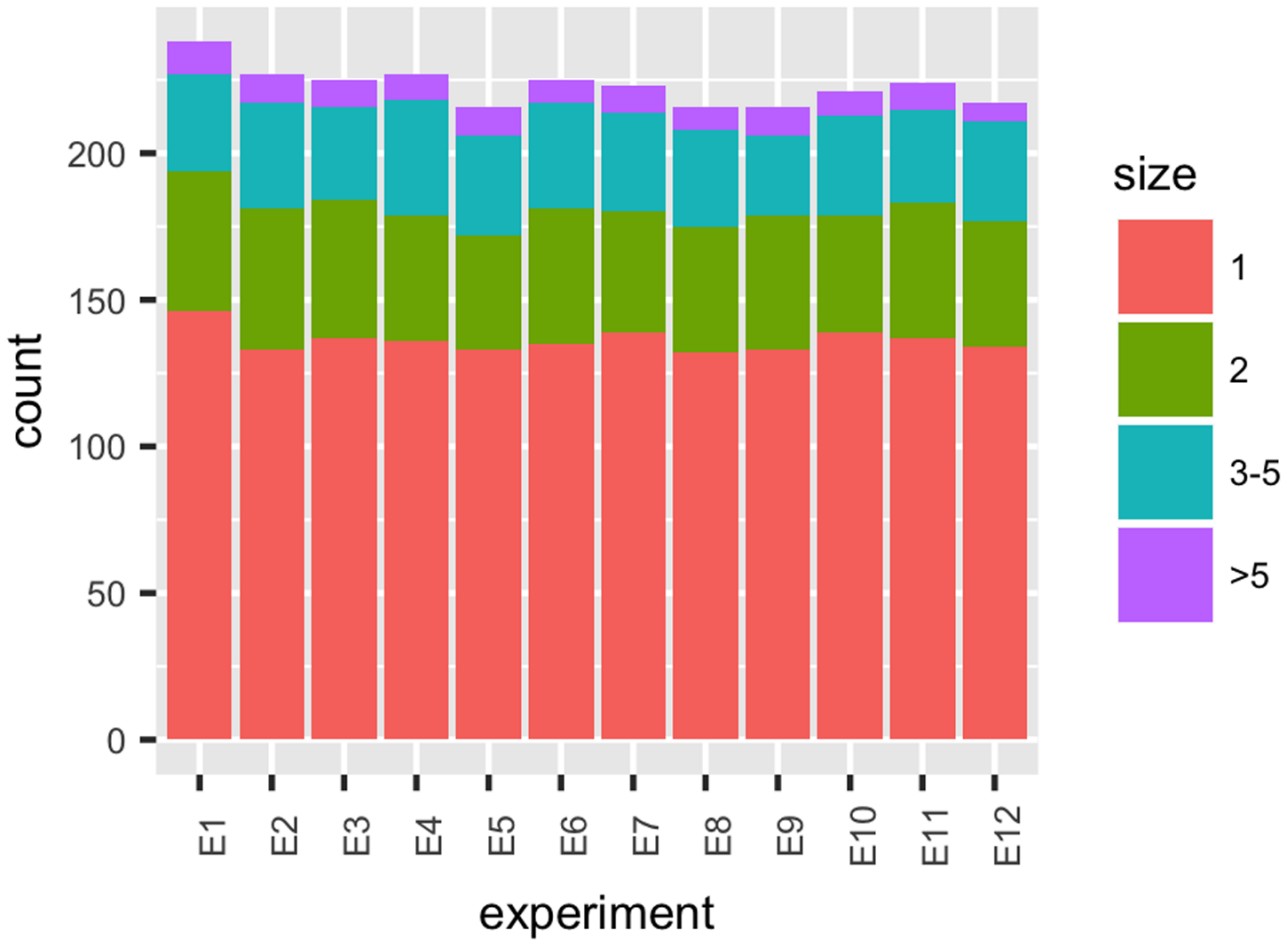
Sizes of protein groups within experimental runs. Sizes of the local groups identified by Proteome Discoverer within each of the 12 spectral count-based experiments in Dataset B.

Dataset B consists of 12 lists of protein groups generated by Proteome Discoverer from 12 spectral count-based experiments used to process plasma samples from 12 healthy individuals. Thus, with this dataset, we examine the effect of technical and biological variation in protein identification (see Materials and methods and S2 File). Fig 4 illustrates the sizes of the local protein groups within each experiment. Similar to what was observed for the iTRAQ experiments, on average, 222 protein groups were identified within each experimental run, with 60% of these containing a single protein identifier. To compare the composition of the groups among the 12 experiments, we use the Dice coefficient proposed by Hesse et al. [7] as a measure of similarity between protein groups (defined in Materials and methods). In this study, we use this measure to compare the local protein groups (child groups in Hesse et al.’s terminology) within each experiment with the global groups (parent groups) in the dictionary created by PGCA. The dictionary from these 12 experiments contains a total of 357 global proteins groups.

**Fig 5:**
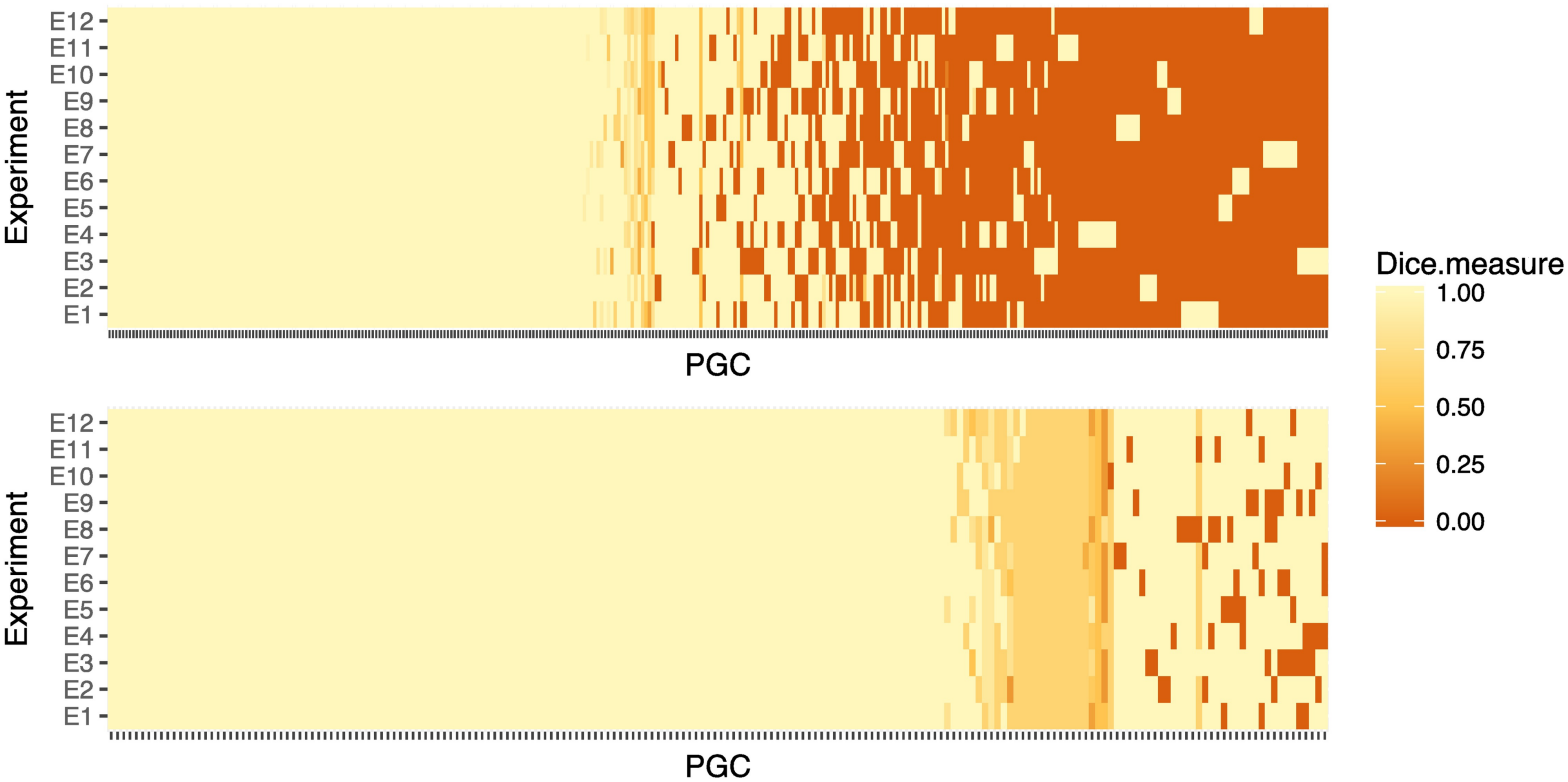
The Dice coefficient to compare lists of protein groups. (A) Dice coefficient to compare the raw protein groups identified within each experiment with the dictionary created by PGCA. (B) Dice coefficient to compare the top-protein of a given group with the set of all top-proteins used to represent the group in other experiments.

We first compare the raw protein groups created by Proteome Discoverer (i.e., the local groups are not refined) with the corresponding global groups in the dictionary created by PGCA. If a global group in the dictionary is not identified in an individual experiment, the intersection between the global and the local group is empty and the Dice coefficient is 0 (dark-orange cell in Fig 5A). If a local group contains the same protein identifiers as the global group (except perhaps for the order of the proteins in the group), the intersection is the same group and the Dice coefficient is 1 (light-yellow cells in Fig 5A). Lastly, if only a subset of the protein identifiers in the global group are identified in an individual experiment, the intersection between the local and global group equals the local group and the Dice coefficient is smaller than 1 (represented by a range of colours between dark-orange and light-yellow in Fig 5A). As expected from a mass spectrometry experiment, 22% of the 357 global groups were identified in only one experiment (dark-orange columns with only one light-yellow cell at the right in Fig 5A) and 45% were identified in all the experiment, although perhaps with a different structure (light yellow and light orange columns to the left in Fig 5A).

However, the Dice coefficient is a measure of similarity between two unordered sets and cannot capture changes in the structure of the groups. For example, among the 45% protein groups identified in all 12 experiments, 23% of them were identified with different top-proteins among the different experiments. Since it is a common practice to use only the top-protein of the identified groups to link lists from multiple experiments, Fig 5B illustrates the changes in the top-protein among groups from different experiments. In this comparison, only the top-protein is retained from each local group and compared against the set of all the top-proteins appearing in related groups (i.e., the union of all top-proteins in the local groups). As before, a Dice coefficient equal to 0 (dark-orange cells in Fig 5B) indicates that the group was not identified in the experiment. A Dice coefficient equal to 1 (light-yellow cells in Fig 5B) indicates that there is a unique top-protein representing all local groups (the local groups may not be identical but they are all represented by the same top-protein). A Dice coefficient smaller than 1 (range of colours between dark-orange and light-yellow in Fig 5B) indicates that different top-proteins were used to represent the group across experiments (i.e., the parent group containing all possible top-proteins has more protein identifiers than each individual group). This study shows that similar results are observed when different biological samples are processed within and across experiments (see also next section), showing that the observed variation in protein identification is dominated by the technical variation.

### Application to biomarkers studies

In general in a biomarker study, the proteins identified in (almost) all experimental runs are analyzed to compare protein levels from different subject groups (e.g., diseased vs healthy patients). As previously discussed, two main tasks are required before starting this discovery analysis. First, the protein identifiers in each identified group (or a selected subset of these) need to be translated into a unique protein group code (PGC) identifier for each experimental run. Second, the assigned group code is used to link the groups among runs and to retain those consistently detected in all (or almost all) runs for subsequent statistical analyses. Importantly, these steps are required even if only the top-protein in each group will be retained, since the lists of top-proteins also vary, both in content and order, across runs. Using different criteria to link the lists of identified proteins results in different sets of proteins to be analyzed. For example, the group illustrated in Fig 2 can be compared in all 3 runs only when groups are linked using the PGC assigned by PGCA-All. Alternatively, if only the top-protein is used, then IPI00215894.1 (UniProt P01042-2) is observed in only the first file; IPI00797833.3 (almost identical to UniProt P01042-2) is observed in the last two files, and IPI00032328.2 (UniProt P01042-1) is observed in files 1 and 3. This may result in losing certain proteins from subsequent analyses if their identification rate across all runs in the study is not large enough.

#### Heart transplantation data

**Fig 6:**
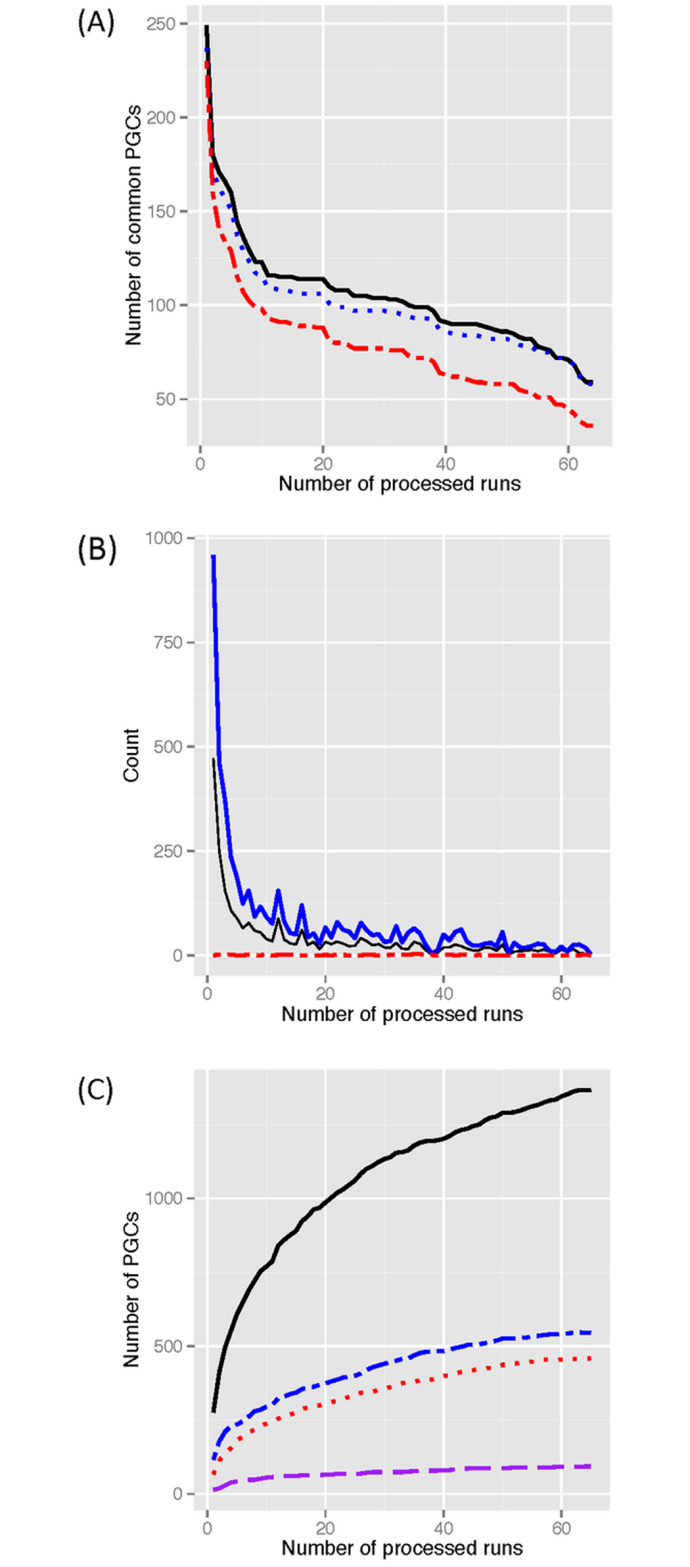
Mapping results according to PGCA. (A) Number of consistently identified protein group codes (common PGCs on y-axis) linked by different PGCA approaches (PGCA-All in solid black, PGCA-topGene in dotted blue, and PGCA-topIdentifier in dotted-dashed red) after a stepwise incorporation of each experiment (x-axis). (B) Number of new protein identities (thick-solid line), of new global protein groups (thin-solid line), and of mergers (dotted line) after a stepwise processing of each experiment. (C) Number of global protein groups in the sequential mappings for different ranges of group sizes (single protein groups in solid black line, groups with 2 proteins in dash-dotted blue line, groups with 3 to 5 proteins in dotted red line, groups with more than 5 proteins in dashed purple line).

The Biomarkers in Transplantation (BiT) team conducted a large biomarker study seeking protein markers of acute rejection in cardiac and renal transplantation using plasma samples [19, 20]. Fig 6A illustrates the number of protein group *codes* that are consistently identified in different experimental runs of the heart transplantation study (dataset C in S3 File). To ease the visualization of the results, the 65 files are incorporated in the analysis one-at-a-time in decreasing order according to the total number of protein groups within each file. As expected, the number of codes identified in all runs decreases as the number of runs increases for all three approaches. This result characterizes the lack of consistency in the identification of proteins previously observed in mass spectrometry data [3, 21]. The distance between the solid and the other lines shows that part of this lack of consistency is explained by changes in the composition of the identified protein groups as previously observed in dataset A. In small studies (1 to 10 runs, Fig 6A), the number of codes consistently identified in all runs increases, on average, from 134 to 162 if the code assigned by PGCA-All is used to link the identifications across runs (20% increment), instead of using the top-protein. Similar patterns are observed in medium studies (10 to 30 runs) with a 33% average increment and in large studies (more than 30 runs) with a 50% average increment in the number of common codes among runs (e.g., from 35 to 59 common codes among 65 runs). Thus, incorporating the information contained in the detected protein groups and allowing these groups to slightly vary their composition across runs may reduce the impact of the lack of identification consistency in subsequent analyzes.

One goal of this biomarker study was to identify potential proteomic markers of acute cardiac allograph rejection. A total of 17 iTRAQ experiments were used to process 6 plasma samples from patients with acute heart rejection (AR) and 12 from patients without rejection (NR). Using PGCA-All in this study to link the protein groups identified across different runs enabled the analysis of 127 protein groups, of which 61 were not represented by the same top-identifier in all runs. Some of the candidate markers with statistically significant differential protein levels between AR and NR groups were represented by different top-proteins across the 17 iTRAQ runs and would have been missed if only the top-protein was used to represent each protein group. For example, Isoform 1 of Sex hormone-binding globulin protein (SHBG) was the top-protein in 13 out of 17 iTRAQ experiments in the study and Isoform 2 of the same protein was the top-protein in the 4 remaining experiments. In particular this candidate marker (robust limma test p-value of 0.03, Wilcoxon p-value of 0.06) was validated using multiple reaction monitoring coupled with mass spectrometry (MRM assay) showing differential protein levels between the AR and NR groups (with Wilcoxon p-value of 0.03). All data from this study is available in S4 File.

#### Muscular dystrophy data

The Jain Foundation with MRM Proteomics and PROOF Centre of Excellence is conducting a proteomics discovery study of blood-based biomarkers associated with skeletal muscle function. Using PGCA-All to link the protein groups identified across different runs enabled the analysis of 131 protein groups identified in more than 75% of the samples, of which 26 were not represented by the same top-identifier in all runs. Of the 26 that would have been missed if only the top-protein were used to link the identified groups, 9 had a false discovery rate (FDR) below 10% in a statistical analysis that compares the protein levels of LGMD2B (n = 50) versus control samples (n = 25). The difference in groups identifications are shown in S5 File. Although these results remain to be validated in an independent cohort, identifying potential candidate markers is crucial in the discovery phase before moving to the validation stage [20].

### Variation in PGCA’s mapping

PGCA creates a mapping of local to global protein groups by comparing the composition of the local protein groups identified within each experimental run. Using a unique global protein group code to represent all the proteins identified in connected local groups enables linking related groups from multiple experimental runs, even if these groups slightly change their composition across runs. Noteworthy, the mapping created by PGCA is data driven and depends on the list of local protein groups identified within each experimental run. Thus, if new experiments are added to a study, the current mapping needs to be updated accordingly (see Fig 2). To study the variation in PGCA’s mapping, each experiment from the dataset C was sequentially processed by PGCA and the resulting mapping was examined. Three possible changes may occur simultaneously or in isolation to a mapping being updated: *i)* new global protein groups are added, *ii)* new protein identities are added to existing global protein groups, and *iii)* existing global protein groups are merged due to the incorporation of connecting groups in the mapping.

Fig 6B illustrates the contribution of these components of variation in the mapping. For simplicity, only results from PGCA-All are shown as similar results were observed for PGCA-topGene and the figure is trivial for PGCA-topIdentifier. We note that increments in the number of new accession numbers (thick-solid line) result in similar increments in the number of new groups (thin-solid line). Thus, most of the new protein identities are non-redundant creating new global groups. The distance between both solid lines corresponds to new protein identities joining existing groups and/or new protein identities from the new non-singleton groups. As the number of processed experimental runs increases, both lines get closer together showing that changes in the mapping are mainly due to the identification of new groups of size 1 instead of a reconfiguration of the existing groups (i.e., addition of new proteins to existing groups and/or merger of groups). The number of mergers at each step (dotted line) remains always below 3 indicating that local groups are consistently connected across runs.

A complementary measure examined is the growth in the total number of global protein groups in the mappings for different ranges of group sizes (Fig 6C). Similar results were observed for PGCA-topGene. Groups with a unique identifier (size 1) are the ones growing faster. The number of groups with sizes between 2 and 5 are also growing but at a lower pace and remains almost constant after processing 45 experiments. Overall, results demonstrate the stabilization of the created mapping. The observed variation in protein identifications and the non-negligible percentage of proteins identified in isolation in each run characterize most mass spectrometry identification lists.

### Properties

This Section presents some properties of PGCA with proof outlines given in S1 Appendix. As these properties hold for the three PGCA approaches described before, we uniformly refer to PGCA. We give more details on the notation used in the Materials and Method section.

Each time a new file is processed, the updated mapping results in a comprehensive list of global protein groups, each with a protein group code, that can be used to link groups across experimental runs. The following results demonstrate that the global groups in the mapping are disjoint and that each was derived from connected local groups. The corollaries at the end of this Section show two results with important practical utility. First, the same mapping is obtained regardless of the order in which files are processed (i.e., order invariance). Second, a current mapping can be easily updated every time that new files are incorporated into a study without the need of reprocessing all the data (i.e., incremental).

**Definition 1** *Let* A *be a set of protein groups*. *Given two groups* $B_{1}\in A$ *and* $B_{2}\in A$, *B*_1_ *and* *B*_2_ *are connected if either B*_1_ ∩ *B*_2_ ≠ ∅ *or there exist a connector set* $\bar{B}=\cup_{j=3}^{K}B_{j}$, *with all B*_*j*_ *in* A, *such that:*

1. $B_{i}\cap \bar{B}\neq \emptyset$ *for* *i* = 1, 2, *and*
2. $\bar{B}$ *can not be partitioned into two disjoint collections of sets in* A.

*Further*, A *is connected if all groups* B∈A *are connected*.

**Lemma 1** *Let L be the set of local groups from a file F (i.e., L = CreateLocalGroups(F)), G be the set of global groups from a dictionary D, and* B=L∪G. *Let* $\tilde{D}=UpdateDictionary\left(D,L\right)$ *be the output dictionary from the UdpdateDictionary algorithm*. *Given B, a set of accession numbers from those in* B, *and* A={E∈B such that B∩E=E}, *B is a global group in* $\tilde{D}$ *if and only if* A *is non-empty and connected, and* B∩E=∅,∀E∈B\A.

From Lemma 1, it easily follows that any global group *B* in $\tilde{D}$ can be written as the union of all connected group in B that are included in *B*.

**Theorem 1** *Let L_i_ be the set of local groups from an input file F_i_ (i.e., L_i_ = CreateLocalGroups(F_i_)) for i* = 1, …, *nFiles, and* $L_{nFiles}=\cup_{i=1}^{nFiles}L_{i}$ *be the universe of all these local groups*. *Starting from an empty dictionary D*_0_, *let D_nFiles_ = PGCA*(*D*_0_, *F*_1_, …, *F*_*nFiles*_) *be the output dictionary from input files F*_*i*_, *i* = 1, …, *nFiles*. *Given B a set of accession numbers from those in* $L_{nFiles}$ *and* $A=\{E\in L_{nFiles}:\;B\cap E=E\}$, *B is a global group in D*_*nFiles*_ *if and only if* A *is non-empty and connected, and* $B\cap E=\emptyset ,\;\forall E\in L_{nFiles}\backslash A$.

**Corollary 1** *PGCA is order invariant, i.e., the order in which the files F*_1_, …, *F*_*nFiles*_ *are input in PGCA do not change the general groups in the output D*_*nFiles*_.

**Corollary 2** *PGCA is incremental, i.e., using the same notation as in Theorem 1*,

$$\begin{array}{lll} D_{n+m} & = & UpdateDictionary\left(D_{0},\left\{L_{1},\ldots ,L_{n+m}\right\}\right)\\ & = & UpdateDictionary\left(D_{n},\left\{L_{n+1},\ldots ,L_{n+m}\right\}\right) \end{array}$$

## Materials and methods

This section describes the datasets used to demonstrate the properties of our algorithm. We also present all formal details of PGCA (see Algorithm 1) and its three main algorithms, CreateLocalGroups, UpdateDictionary, and CodingProteinGroups (see Algorithms 2, 3, and 4, respectively).

### Datasets

We use 4 different datasets to examine the protein inference problem when multiple experimental runs are required to process all samples in a study. Dataset A consists of 4 experimental runs used to process 16 technical replicates of a pool of plasma from 16 healthy individuals processed by iTRAQ-MALDI-TOF/TOF methodology (see S1 File). Dataset B consists of 12 spectral count-based experiments used to identify and quantitate proteins in plasma samples from 12 healthy individuals (see S2 File). Dataset C consists of 65 iTRAQ runs used to process 195 samples from heart transplant patients (see S3 and S4 Files). Datasets A to C belong to the Biomarkers in Transplantation (BiT) initiative and PROOF Centre of Excellence. Dataset D belongs to the Jain Foundation and consists of 11 iTRAQ runs used to process 50 limb-girdle muscular dystrophy type 2 B (LGMD2B) and 25 healthy controls (see S5 File). All these datasets are based on studies approved by the Human Research Ethics Board of the University of British Columbia. All patients enrolled in these studies signed consent forms. Similar experimental and data processing methods were used to process the plasma samples in all datasets, which are described in detail in [19, 20], except for those in dataset B which are raw (non-depleted) plasma samples without any isobaric label added. The raw MS/MS data from datasets A and C were processed using ProteinPilot software v3.0 with the integrated Paragon^™^ Search and Pro Group^™^ algorithms [12], searching against International Protein Index (IPI HUMAN v3.39) database [22]. Datasets B and D were processed using Thermo Proteome Discoverer 1.4 and MASCOT, searching against UniProt database (October 2016 and 2014 release, respectively). Although we can not reprocess some of the data used in this work, we note that the protein inference problem is inherent in tandem mass spectrometry techniques regardless of the proteomics technology and the standard databases used. All codes were written in R (http://www.r-project.org). A library with an associated Vignette to implement PGCA algorithm is publicly available from Bioconductor (http://www.bioconductor.org).

### Dice coefficient

Hesse et al. [7] proposed using the Dice coefficient to compare list of protein groups identified from different experiments (child context) with a parent list generated as the union of all individual lists. The Dice coefficient is a measure of similarity between two (unordered) sets and is defined by 2|*A* ∩ *B*|/(|*A*| + |*B*|), where |.| denotes the number of elements in the set. Note that if the sets *A* and *B* do not share any elements, then the Dice coefficient equals 0. If the sets *A* and *B* have the same elements, then the dice measure equals 1. However, this measure can not capture differences in the order of the elements of the sets. For example, if two protein groups contain the same set of protein identifiers then the Dice coefficient equals 1, even if each group is represented by a different top-protein in different experiments.

### Notation

As a result of processing MS/MS data, most proteomics software generate protein summaries containing quantitative and qualitative information of the sample(s) analyzed within each run. PGCA uses these protein summaries as input files, here named *F*_1_, …, *F*_*nFiles*_, with *nFiles* being the total number of input files. PGCA works on the assumption that the following variables are available within each protein summary, or easily generated as a result of processing MS/MS data:

1. Group identifier (N): an integer used to define (local) protein groups within each experimental run
2. Top-protein identifier (TopID): an identifier of the top protein identification within each protein group
3. Accession number (Acc): an identifier from a proteomic database for each identified protein
4. Protein name (Prot): the protein name in the database matching to the accession number
5. Gene symbol (Gene): gene(s) matching to the identified protein
6. $\bar{\text{Rest}}$: additional information from the input file (e.g., confidence of identification, percentage of coverage, protein (relative) levels).

Further, we assume that each input file has the variables defined above as columns, i.e., *F*_*k*_ has entries <N, TopID, Acc, Prot, Gene, $\bar{\text{Rest}}$ >.

PGCA creates a dictionary, *D*_*nFiles*_, adding a protein group code (PGC) to each global group created from *F*_1_, …, *F*_*nFiles*_. The subscript *nFiles* is used to indicate how many files were used to create the dictionary. In practice, the set of files used can range from the total number of files in a study to specific files used to process data to answer a particular question.

The resulting dictionary is used to recode each entry of *F*_1_, …, *F*_*nFiles*_ and thus generate *nFiles* output files, denoted as *CF*_1_, …, *CF*_*nFiles*_. Each entry in *CF*_*k*_ is of the form <PGC, N, TopID, Acc, Prot, Gene, $\bar{\text{Rest}}$ >, where PGC is the code corresponding to the protein accession number (Acc) in the dictionary.

In PGCA, we define the *j*-th local group $LG_{j}^{k}$ in *F*_*k*_ as the set of $n_{j}^{k}$ accession numbers from entries in *F*_*k*_ with identical group identifier *j*, i.e., $LG_{j}^{k}=\left\{{\text{Acc}}_{i}\in F_{k}:{\text{N}}_{i}=j\right\}$; and *nLocalGroups* the total number of distinct Ns in *F*_*k*_. Similarly, The *j*-th global group *GG*_*j*_ in *D* is defined as the set of *m*_*j*_ accession numbers from entries in a dictionary *D* with identical PGC identifier *j*, i.e., *GG*_*j*_ = {Acc_*i*_ ∈ *D*: PGC_*i*_ = *j*}; and *nGlobalGroups* the total number of distinct PGCs in *D*. To simplify the notation, we assume that all groups are numbered in consecutive order, since one can always re-name existing groups.

Each algorithm is presented as a mathematical function, i.e., *f*(*x*) = *y*, to state that the algorithm *f* transforms the input file *x* into the output object *y*.

### Algorithm

We only present PGCA-All in detail since PGCA-topGene and PGCA-topIdentifier only differ from it in the CreateLocalGroups algorithm within each. In particular, each local group in the LocalGroupSet created by the CreateLocalGroups algorithm in PGCA-topGene retains only those entries from the full local group with identical rank and Gene element as those of the top protein identification. Similarly, CreateLocalGroups algorithm in PGCA-topIdentifier retains only the top entry. Thus, the local groups created by these algorithms are subsets of those created by the PGCA-All (see Fig 3).

**Algorithm 1:** PGCA

 **Input:** *F*_1_, …, *F*_*nFiles*_

 **Output:** *D*_*nFiles*_, *CF*_1_, …, *CF*_*nFiles*_

1 Let *D*_0_ be an empty dictionary

2 **for** 1 ≤ *k* ≤ *nFiles* **do**

3  CreateLocalGroups(*F*_*k*_) = *LocalGroupSet*_*k*_

4  UpdateDictionary(*LocalGroupSet*_*k*_, *D*_*k*−1_) = *D*_*k*_

5 **for** 1 ≤ *k* ≤ *nFiles* **do**

6  CodingProteinGroups(*D*_*nFiles*_, *F*_*k*_) = *CF*_*k*_. ∎

**Algorithm 2:** CreateLocalGroups

 **Input:** *F*_*k*_, the *k*-th input file

 **Output:** $LocalGroupSet_{k}=\left\{LG_{j}^{k};j=1,\ldots ,nLocalGroups_{k}\right\}$

1 **for** 1 ≤ *j* ≤ *nLocalGroups*_*k*_ **do**

2  create $LG_{j}^{k}=\left\{{\text{Acc}}_{i}\in F_{j}^{k}:{\text{N}}_{i}=j\right\}$

3 define $LocalGroupSet_{k}=\left\{LG_{j}^{k};j=1\;\ldots ,nLocalGroups_{k}\right\}$. ∎

**Algorithm 3:** UpdateDictionary

 **Input:** A dictionary *D* and a LocalGroupSet

 **Output:** An updated dictionary $\tilde{D}$

 /* Create a GlobalGroupSet */

1 **if** *D* = *D*_0_ **then return** *GlobalGroupSet* = ∅;

2 **else**

3 **for** 1 ≤ *j* ≤ *nGlobalGroups* **do**

4  create *GG*_*j*_ = {Acc_*i*_ ∈ *D*: PGC_*i*_ = *j*}

5 define *GlobalGroupSet* = {*GG*_*j*_; *j* = 1…, *nGobalGroups*};

 /* Update the GlobalGroupSet */

6 create B=GlobalGroupSet∪LocalGroupSet

 /* Merge overlapping and connected groups to a fixpoint */

7 **do**

8  take B∈B

9  **repeat**

   /* inner loop to get a fixpoint */

10   compute E={E∈B:E∩B≠∅}

11   **if** E=∅ **then**

12    *B* is marked “done”

13   **else**

14    $B\leftarrow B\cup_{E\in E}E$;

15    Update B to remove all E∈E

16  **until** E=∅;

17 **while** ∃B∈B:B *is not marked “done”*;

 /* Update D */

18 assign a distinct PGC to each B∈B, i.e., 〈*PGC*_*B*_, *B*〉

19 create $\tilde{D}=\left\{\langle PGC_{B},B\rangle :\;B\in B\right\}$ ∎

**Algorithm 4:** CodingProteinGroups

 **Input:** *F*_*k*_ and the corresponding *D*

 **Output:** *CF*_*k*_

1 **for** *each entries* *a* =< *N*_*a*_, *TopID*_*a*_, *Acc*_*a*_, *Prot*_*a*_, *Gene*_*a*_, ${\bar{Rest}}_{a}$ > *in* *F*_*k*_ **do**

2  find the unique entry in *D*, such that Acc = Acc_*a*_

3  let PGC_*a*_ be the protein group code corresponding to Acc_*a*_ in *D*

 /* code entries in files */

4 code entry *a* as *c*_*a*_ =< PGC_*a*_, N_*a*_, TopID_*a*_, Acc_*a*_, Prot_*a*_, Gene_*a*_, ${\bar{\text{Rest}}}_{a}$ > *CF*_*k*_ = {*c*_*a*_; for all *a* entries in *F*_*k*_} ∎

## Conclusions

PGCA offers a pre-processing tool required in many typical workflows of quantitative proteomics data analysis. PGCA allows linking lists of identified proteins organized by groups without making assumption about the presence or the order of proteins within each group. PGCA extends the idea of protein groups to a multiple-experimental setting enabling the connection and comparability of related groups of proteins. The user can tune the degree of connectivity by choosing different criteria within PGCA.

Comparing the list of detected groups instead of individual proteins identities retains (all) the information contained in the groups maintaining the lists of non-redundant protein identities created within runs. In most cases, the comparison of these proteins is supported by the similarity of their sequences and the overlap among the lists of identified peptides from multiple experimental runs. Although the protein inference problem is well understood in the proteomics community, in this paper we characterize this problem and quantify its impact in subsequent quantitative and qualitative analyses based on multiple experimental runs.

## Supporting information

S1 Fig Protein group example from dataset C in Fig 2. *i*. Protein IPI00215894.1 is in IPI.HUMAN.v3.87 database (last version of IPI), its accession number in UniProt is P01042-2, and is the isoform of LMW of Kininogen-1 (KNG1). Protein IPI00797833.3 differs from IPI00215894.1 by only one amino acid and was removed from IPI.HUMAN.v3.87; *ii*. Protein IPI00032328.2 (in IPI.HUMAN.v3.87) corresponds to P01042-1 in UniProt, and is the isoform HMW of KNG1; *iii*. Alignment of the 3 disRnct proteins in the protein group by Clustal Omega v1.2.2.
(PDF)

S2 Fig Protein group example from dataset C in Fig 3. Alignment of the 4 distinct proteins in the protein group by Clustal Omega v1.2.2.
(PDF)

S1 Appendix Proof of properties. (PDF)

S1 File Data files in dataset A. 4 iTRAQ experiments with technical replicates.
(ZIP)

S2 File Data files in dataset B. 12 spectral count-based experiments from 12 plasma samples.
(ZIP)

S3 File Data files in dataset C. 65 iTRAQ experiments used to process samples from the BiT Heart Cohort.
(ZIP)

S4 File Data files used in the heart transplantation biomarkers study. Subset from dataset C.
(ZIP)

S5 File Data files in dataset D. Protein identification information from the muscular dystrophy biomarkers study.
(ZIP)
