# Supplementary material for: PGCA: An algorithm to link protein groups created from MS/MS data
Source: PLoS One. 2017 May 31;12(5):e0177569. doi: 10.1371/journal.pone.0177569 (PMC5451011; doi:10.1371/journal.pone.0177569)
Supplement: S1 Fig — i. Protein IPI00215894.1 is in IPI.HUMAN.v3.87 database (last version of IPI), its accession number in UniProt is P01042-2, and is the isoform of LMW of Kininogen-1 (KNG1). Protein IPI00797833.3 differs from IPI00215894.1 by only one amino acid and was removed from IPI.HUMAN.v3.87; ii. Protein IPI00032328.2 (in IPI.HUMAN.v3.87) corresponds to P01042-1 in UniProt, and is the isoform HMW of KNG1; iii. Alignment of the 3 disRnct proteins in the protein group by Clustal Omega v1.2.2. (PDF) [file pone.0177569.s001.pdf]

S1 Fig. *i*.

```
CLUSTAL O(1.2.2) multiple sequence alignment


IPI:IPI00215894.1|SWISS-PROT:P01042-2|ENSEMBL:ENSP00000287611|Gene_Symbol=KNG1      MKLITILFLCSRLLLSLTQESQSEEIDCNDKDLFKAVDAALKKYNSQNQSNNQFVLYRIT
sp|P01042-2|KNG1_HUMAN                                                              MKLITILFLCSRLLLSLTQESQSEEIDCNDKDLFKAVDAALKKYNSQNQSNNQFVLYRIT
IPI:IPI00797833.3|Gene_Symbol=KNG1                                                  MKLITILFLCSRLLLSLTQESQSEEIDCNDKDLFKAVDAALKKYNSQNQSNNQFVLYRIT
                                                                                    ************************************************************

IPI:IPI00215894.1|SWISS-PROT:P01042-2|ENSEMBL:ENSP00000287611|Gene_Symbol=KNG1      EATKTVGSDTFYSFKYEIKEGDCPVQSGKTWQDCEYKDAAKAATGECTATVGKRSSTKFS
sp|P01042-2|KNG1_HUMAN                                                              EATKTVGSDTFYSFKYEIKEGDCPVQSGKTWQDCEYKDAAKAATGECTATVGKRSSTKFS
IPI:IPI00797833.3|Gene_Symbol=KNG1                                                  EATKTVGSDTFYSFKYEIKEGDCPVQSGKTWQDCEYKDAAKAATGECTATVGKRSSTKFS
                                                                                    ************************************************************

IPI:IPI00215894.1|SWISS-PROT:P01042-2|ENSEMBL:ENSP00000287611|Gene_Symbol=KNG1      VATQTCQITPAEGPVVTAQYDCLGCVHPISTQSPDLEPILRHGIQYFNNNTQHSSLFMLN
sp|P01042-2|KNG1_HUMAN                                                              VATQTCQITPAEGPVVTAQYDCLGCVHPISTQSPDLEPILRHGIQYFNNNTQHSSLFMLN
IPI:IPI00797833.3|Gene_Symbol=KNG1                                                  VATQTCQITPAEGPVVTAQYDCLGCVHPISTQSPDLEPILRHGIQYFNNNTQHSSLFMLN
                                                                                    ************************************************************

IPI:IPI00215894.1|SWISS-PROT:P01042-2|ENSEMBL:ENSP00000287611|Gene_Symbol=KNG1      EVKRAQRQVVAGLNFRITYSIVQTNCSKENFLFLTPDCKSLWNGDTGECTDNAYIDIQLR
sp|P01042-2|KNG1_HUMAN                                                              EVKRAQRQVVAGLNFRITYSIVQTNCSKENFLFLTPDCKSLWNGDTGECTDNAYIDIQLR
IPI:IPI00797833.3|Gene_Symbol=KNG1                                                  EVKRAQRQVVAGLNFRMTYSIVQTNCSKENFLFLTPDCKSLWNGDTGECTDNAYIDIQLR
                                                                                    ****************:*******************************************

IPI:IPI00215894.1|SWISS-PROT:P01042-2|ENSEMBL:ENSP00000287611|Gene_Symbol=KNG1      IASFSQNCDIYPGKDFVQPPTKICVGCPRDIPTNSPELEETLTHTITKLNAENNATFYFK
sp|P01042-2|KNG1_HUMAN                                                              IASFSQNCDIYPGKDFVQPPTKICVGCPRDIPTNSPELEETLTHTITKLNAENNATFYFK
IPI:IPI00797833.3|Gene_Symbol=KNG1                                                  IASFSQNCDIYPGKDFVQPPTKICVGCPRDIPTNSPELEETLTHTITKLNAENNATFYFK
                                                                                    ************************************************************

IPI:IPI00215894.1|SWISS-PROT:P01042-2|ENSEMBL:ENSP00000287611|Gene_Symbol=KNG1      IDNVKKARVQVVAGKKYFIDFVARETTCSKESNEELTESCETKKLGQSLDCNAEVYVVPW
sp|P01042-2|KNG1_HUMAN                                                              IDNVKKARVQVVAGKKYFIDFVARETTCSKESNEELTESCETKKLGQSLDCNAEVYVVPW
IPI:IPI00797833.3|Gene_Symbol=KNG1                                                  IDNVKKARVQVVAGKKYFIDFVARETTCSKESNEELTESCETKKLGQSLDCNAEVYVVPW
                                                                                    ************************************************************

IPI:IPI00215894.1|SWISS-PROT:P01042-2|ENSEMBL:ENSP00000287611|Gene_Symbol=KNG1      EKKIYPTVNCQPLGMISLMKRPPGFSPFRSSRIGEIKEETTSHLRSCEYKGRPPKAGAEP
sp|P01042-2|KNG1_HUMAN                                                              EKKIYPTVNCQPLGMISLMKRPPGFSPFRSSRIGEIKEETTSHLRSCEYKGRPPKAGAEP
IPI:IPI00797833.3|Gene_Symbol=KNG1                                                  EKKIYPTVNCQPLGMISLMKRPPGFSPFRSSRIGEIKEETTSHLRSCEYKGRPPKAGAEP
                                                                                    ************************************************************

IPI:IPI00215894.1|SWISS-PROT:P01042-2|ENSEMBL:ENSP00000287611|Gene_Symbol=KNG1      ASEREVS
sp|P01042-2|KNG1_HUMAN                                                              ASEREVS
IPI:IPI00797833.3|Gene_Symbol=KNG1                                                  ASEREVS
                                                                                    *******
```

S1 Fig. *ii*.

```
CLUSTAL O(1.2.2) multiple sequence alignment


IPI:IPI00032328.2|SWISS-PROT:P01042-1|ENSEMBL:ENSP00000265023|Gene_Symbol=KNG1      MKLITILFLCSRLLLSLTQESQSEEIDCNDKDLFKAVDAALKKYNSQNQSNNQFVLYRIT
sp|P01042|KNG1_HUMAN                                                                MKLITILFLCSRLLLSLTQESQSEEIDCNDKDLFKAVDAALKKYNSQNQSNNQFVLYRIT
                                                                                    ************************************************************

IPI:IPI00032328.2|SWISS-PROT:P01042-1|ENSEMBL:ENSP00000265023|Gene_Symbol=KNG1      EATKTVGSDTFYSFKYEIKEGDCPVQSGKTWQDCEYKDAAKAATGECTATVGKRSSTKFS
sp|P01042|KNG1_HUMAN                                                                EATKTVGSDTFYSFKYEIKEGDCPVQSGKTWQDCEYKDAAKAATGECTATVGKRSSTKFS
                                                                                    ************************************************************

IPI:IPI00032328.2|SWISS-PROT:P01042-1|ENSEMBL:ENSP00000265023|Gene_Symbol=KNG1      VATQTCQITPAEGPVVTAQYDCLGCVHPISTQSPDLEPILRHGIQYFNNNTQHSSLFMLN
sp|P01042|KNG1_HUMAN                                                                VATQTCQITPAEGPVVTAQYDCLGCVHPISTQSPDLEPILRHGIQYFNNNTQHSSLFMLN
                                                                                    ************************************************************

IPI:IPI00032328.2|SWISS-PROT:P01042-1|ENSEMBL:ENSP00000265023|Gene_Symbol=KNG1      EVKRAQRQVVAGLNFRITYSIVQTNCSKENFLFLTPDCKSLWNGDTGECTDNAYIDIQLR
sp|P01042|KNG1_HUMAN                                                                EVKRAQRQVVAGLNFRITYSIVQTNCSKENFLFLTPDCKSLWNGDTGECTDNAYIDIQLR
                                                                                    ************************************************************

IPI:IPI00032328.2|SWISS-PROT:P01042-1|ENSEMBL:ENSP00000265023|Gene_Symbol=KNG1      IASFSQNCDIYPGKDFVQPPTKICVGCPRDIPTNSPELEETLTHTITKLNAENNATFYFK
sp|P01042|KNG1_HUMAN                                                                IASFSQNCDIYPGKDFVQPPTKICVGCPRDIPTNSPELEETLTHTITKLNAENNATFYFK
                                                                                    ************************************************************

IPI:IPI00032328.2|SWISS-PROT:P01042-1|ENSEMBL:ENSP00000265023|Gene_Symbol=KNG1      IDNVKKARVQVVAGKKYFIDFVARETTCSKESNEELTESCETKKLGQSLDCNAEVYVVPW
sp|P01042|KNG1_HUMAN                                                                IDNVKKARVQVVAGKKYFIDFVARETTCSKESNEELTESCETKKLGQSLDCNAEVYVVPW
                                                                                    ************************************************************

IPI:IPI00032328.2|SWISS-PROT:P01042-1|ENSEMBL:ENSP00000265023|Gene_Symbol=KNG1      EKKIYPTVNCQPLGMISLMKRPPGFSPFRSSRIGEIKEETTVSPPHTSMAPAQDEERDSG
sp|P01042|KNG1_HUMAN                                                                EKKIYPTVNCQPLGMISLMKRPPGFSPFRSSRIGEIKEETTVSPPHTSMAPAQDEERDSG
                                                                                    ************************************************************

IPI:IPI00032328.2|SWISS-PROT:P01042-1|ENSEMBL:ENSP00000265023|Gene_Symbol=KNG1      KEQGHTRRHDWGHEKQRKHNLGHGHKHERDQGHGHQRGHGLGHGHEQQHGLGHGHKFKLD
sp|P01042|KNG1_HUMAN                                                                KEQGHTRRHDWGHEKQRKHNLGHGHKHERDQGHGHQRGHGLGHGHEQQHGLGHGHKFKLD
                                                                                    ************************************************************

IPI:IPI00032328.2|SWISS-PROT:P01042-1|ENSEMBL:ENSP00000265023|Gene_Symbol=KNG1      DDLEHQGGHVLDHGHKHKHGHGHGKHKNKGKKNGKHNGWKTEHLASSSEDSTTPSAQTQE
sp|P01042|KNG1_HUMAN                                                                DDLEHQGGHVLDHGHKHKHGHGHGKHKNKGKKNGKHNGWKTEHLASSSEDSTTPSAQTQE
                                                                                    ************************************************************

IPI:IPI00032328.2|SWISS-PROT:P01042-1|ENSEMBL:ENSP00000265023|Gene_Symbol=KNG1      KTEGPTPIPSLAKPGVTVTFSDFQDSDLIATMMPPISPAPIQSDDDWIPDIQIDPNGLSF
sp|P01042|KNG1_HUMAN                                                                KTEGPTPIPSLAKPGVTVTFSDFQDSDLIATMMPPISPAPIQSDDDWIPDIQIDPNGLSF
                                                                                    ************************************************************

IPI:IPI00032328.2|SWISS-PROT:P01042-1|ENSEMBL:ENSP00000265023|Gene_Symbol=KNG1      NPISDFPDTTSPKCPGRPWKSVSEINPTTQMKESYYFDLTDGLS
sp|P01042|KNG1_HUMAN                                                                NPISDFPDTTSPKCPGRPWKSVSEINPTTQMKESYYFDLTDGLS
                                                                                    ********************************************
```

S1 Fig. *iii*.

```
CLUSTAL O(1.2.2) multiple sequence alignment


IPI:IPI00789376.4|TREMBL:Q05CF8                             ------------------------------------------------------------
IPI:IPI00215894.1|SWISS-PROT:P01042-2|Gene_Symbol=KNG1      MKLITILFLCSRLLLSLTQESQSEEIDCNDKDLFKAVDAALKKYNSQNQSNNQFVLYRIT
IPI:IPI00032328.2|SWISS-PROT:P01042-1|Gene_Symbol=KNG1      MKLITILFLCSRLLLSLTQESQSEEIDCNDKDLFKAVDAALKKYNSQNQSNNQFVLYRIT


IPI:IPI00789376.4|TREMBL:Q05CF8                             ------------------------------------------------------------
IPI:IPI00215894.1|SWISS-PROT:P01042-2|Gene_Symbol=KNG1      EATKTVGSDTFYSFKYEIKEGDCPVQSGKTWQDCEYKDAAKAATGECTATVGKRSSTKFS
IPI:IPI00032328.2|SWISS-PROT:P01042-1|Gene_Symbol=KNG1      EATKTVGSDTFYSFKYEIKEGDCPVQSGKTWQDCEYKDAAKAATGECTATVGKRSSTKFS


IPI:IPI00789376.4|TREMBL:Q05CF8                             -------MHGNR--GEEEQ-------YEILRGYPDLEPILRHGIQYFNNNTQHSSLFTLN
IPI:IPI00215894.1|SWISS-PROT:P01042-2|Gene_Symbol=KNG1      VATQTCQITPAEGPVVTAQYDCLGCVHPISTQSPDLEPILRHGIQYFNNNTQHSSLFMLN
IPI:IPI00032328.2|SWISS-PROT:P01042-1|Gene_Symbol=KNG1      VATQTCQITPAEGPVVTAQYDCLGCVHPISTQSPDLEPILRHGIQYFNNNTQHSSLFMLN
                                                                   :   .      *       : *    ************************ **

IPI:IPI00789376.4|TREMBL:Q05CF8                             EVKRAQRQVVAGLNFRITYSIVQTNCSKENFLFLTPDCKSLWNGDTGECTDNAYIDIQLR
IPI:IPI00215894.1|SWISS-PROT:P01042-2|Gene_Symbol=KNG1      EVKRAQRQVVAGLNFRITYSIVQTNCSKENFLFLTPDCKSLWNGDTGECTDNAYIDIQLR
IPI:IPI00032328.2|SWISS-PROT:P01042-1|Gene_Symbol=KNG1      EVKRAQRQVVAGLNFRITYSIVQTNCSKENFLFLTPDCKSLWNGDTGECTDNAYIDIQLR
                                                            ************************************************************

IPI:IPI00789376.4|TREMBL:Q05CF8                             IASFSQNCDIYPGKDFVQPPTKICVGCPRDIPTNSPELEETLTHTITKLNAENNATFYFK
IPI:IPI00215894.1|SWISS-PROT:P01042-2|Gene_Symbol=KNG1      IASFSQNCDIYPGKDFVQPPTKICVGCPRDIPTNSPELEETLTHTITKLNAENNATFYFK
IPI:IPI00032328.2|SWISS-PROT:P01042-1|Gene_Symbol=KNG1      IASFSQNCDIYPGKDFVQPPTKICVGCPRDIPTNSPELEETLTHTITKLNAENNATFYFK
                                                            ************************************************************

IPI:IPI00789376.4|TREMBL:Q05CF8                             IDNVKKARVQVVAGKKYFIDFVARETTCSKESNEELTESCETKKLGQSLDCNAEVYVVPW
IPI:IPI00215894.1|SWISS-PROT:P01042-2|Gene_Symbol=KNG1      IDNVKKARVQVVAGKKYFIDFVARETTCSKESNEELTESCETKKLGQSLDCNAEVYVVPW
IPI:IPI00032328.2|SWISS-PROT:P01042-1|Gene_Symbol=KNG1      IDNVKKARVQVVAGKKYFIDFVARETTCSKESNEELTESCETKKLGQSLDCNAEVYVVPW
                                                            ************************************************************

IPI:IPI00789376.4|TREMBL:Q05CF8                             EKKIYPTVNCQPLGMISLMKRPPGFSPFRSSRIGEIKEETTSHLRSCEYKGRPPKAGAEP
IPI:IPI00215894.1|SWISS-PROT:P01042-2|Gene_Symbol=KNG1      EKKIYPTVNCQPLGMISLMKRPPGFSPFRSSRIGEIKEETTSHLRSCEYKGRPPKAGAEP
IPI:IPI00032328.2|SWISS-PROT:P01042-1|Gene_Symbol=KNG1      EKKIYPTVNCQPLGMISLMKRPPGFSPFRSSRIGEIKEETTVSPP---------HTSMAP
                                                            *****************************************             ::.  *

IPI:IPI00789376.4|TREMBL:Q05CF8                             ASEREVS-----------------------------------------------------
IPI:IPI00215894.1|SWISS-PROT:P01042-2|Gene_Symbol=KNG1      ASEREVS-----------------------------------------------------
IPI:IPI00032328.2|SWISS-PROT:P01042-1|Gene_Symbol=KNG1      AQDEERDSGKEQGHTRRHDWGHEKQRKHNLGHGHKHERDQGHGHQRGHGLGHGHEQQHGL
                                                            *.:.* .

IPI:IPI00789376.4|TREMBL:Q05CF8                             ------------------------------------------------------------
IPI:IPI00215894.1|SWISS-PROT:P01042-2|Gene_Symbol=KNG1      ------------------------------------------------------------
IPI:IPI00032328.2|SWISS-PROT:P01042-1|Gene_Symbol=KNG1      GHGHKFKLDDDLEHQGGHVLDHGHKHKHGHGHGKHKNKGKKNGKHNGWKTEHLASSSEDS


IPI:IPI00789376.4|TREMBL:Q05CF8                             ------------------------------------------------------------
IPI:IPI00215894.1|SWISS-PROT:P01042-2|Gene_Symbol=KNG1      ------------------------------------------------------------
IPI:IPI00032328.2|SWISS-PROT:P01042-1|Gene_Symbol=KNG1      TTPSAQTQEKTEGPTPIPSLAKPGVTVTFSDFQDSDLIATMMPPISPAPIQSDDDWIPDI


IPI:IPI00789376.4|TREMBL:Q05CF8                             -----------------------------------------------------
IPI:IPI00215894.1|SWISS-PROT:P01042-2|Gene_Symbol=KNG1      -----------------------------------------------------
IPI:IPI00032328.2|SWISS-PROT:P01042-1|Gene_Symbol=KNG1      QIDPNGLSFNPISDFPDTTSPKCPGRPWKSVSEINPTTQMKESYYFDLTDGLS
```
