# Supplementary material for: PGCA: An algorithm to link protein groups created from MS/MS data
Source: PLoS One. 2017 May 31;12(5):e0177569. doi: 10.1371/journal.pone.0177569 (PMC5451011; doi:10.1371/journal.pone.0177569)
Supplement: S2 Fig — Alignment of the 4 distinct proteins in the protein group by Clustal Omega v1.2.2. (PDF) [file pone.0177569.s002.pdf]

S2 Fig.

```
CLUSTAL O(1.2.2) multiple sequence alignment

SP|P14618|KPYM_HUMAN   ---MSKPHSEAGTAFIQTQQLHAAMADTFLEHMCRLDIDSPPITARNTGIICTIGPASRS 57
SP|P14618-2|KPYM_HUMAN ---MSKPHSEAGTAFIQTQQLHAAMADTFLEHMCRLDIDSPPITARNTGIICTIGPASRS 57
SP|P14618-3|KPYM_HUMAN MSPEAQPQRTK-----------------GPQQPCRSPIVKPGLPSFRP-SSCTQPWLTHS 42
TR|Q504U3|Q504U3_HUMAN ---MSKPHSEAGTAFIQTQQLHAAMADTFLEHMCRLDIDSPPS----------------- 40
                           ::*:                      :: **  * .*

SP|P14618|KPYM_HUMAN   VETLKEMIKSGMNVARLNFSHGTHEYHAETIKNVRTATESFASDPILYRPVAVALDTKGP 117
SP|P14618-2|KPYM_HUMAN VETLKEMIKSGMNVARLNFSHGTHEYHAETIKNVRTATESFASDPILYRPVAVALDTKGP 117
SP|P14618-3|KPYM_HUMAN WSTCAAWTLIHHPSQPGTLASSVPLYHAETIKNVRTATESFASDPILYRPVAVALDTKGP 102
TR|Q504U3|Q504U3_HUMAN ------------------------------------------------------------


SP|P14618|KPYM_HUMAN   EIRTGLIKGSGTAEVELKKGATLKITLDNAYMEKCDENILWLDYKNICKVVEVGSKIYVD 177
SP|P14618-2|KPYM_HUMAN EIRTGLIKGSGTAEVELKKGATLKITLDNAYMEKCDENILWLDYKNICKVVEVGSKIYVD 177
SP|P14618-3|KPYM_HUMAN EIRTGLIKGSGTAEVELKKGATLKITLDNAYMEKCDENILWLDYKNICKVVEVGSKIYVD 162
TR|Q504U3|Q504U3_HUMAN ------------------------------------------------------------


SP|P14618|KPYM_HUMAN   DGLISLQVKQKGADFLVTEVENGGSLGSKKGVNLPGAAVDLPAVSEKDIQDLKFGVEQDV 237
SP|P14618-2|KPYM_HUMAN DGLISLQVKQKGADFLVTEVENGGSLGSKKGVNLPGAAVDLPAVSEKDIQDLKFGVEQDV 237
SP|P14618-3|KPYM_HUMAN DGLISLQVKQKGADFLVTEVENGGSLGSKKGVNLPGAAVDLPAVSEKDIQDLKFGVEQDV 222
TR|Q504U3|Q504U3_HUMAN ----------------------------KKGVNLPGAAVDLPAVSEKDIQDLKFGVEQDV 72
                                                   ********************************

SP|P14618|KPYM_HUMAN   DMVFASFIRKASDVHEVRKVLGEKGKNIKIISKIENHEGVRRFDEILEASDGIMVARGDL 297
SP|P14618-2|KPYM_HUMAN DMVFASFIRKASDVHEVRKVLGEKGKNIKIISKIENHEGVRRFDEILEASDGIMVARGDL 297
SP|P14618-3|KPYM_HUMAN DMVFASFIRKASDVHEVRKVLGEKGKNIKIISKIENHEGVRRFDEILEASDGIMVARGDL 282
TR|Q504U3|Q504U3_HUMAN DMVFASFIRKASDVHEVRKVLGEKGKNIKIISKIENHEGVRRFDEILEASDGIMVARGDL 132
                       ************************************************************

SP|P14618|KPYM_HUMAN   GIEIPAEKVFLAQKMMIGRCNRAGKPVICATQMLESMIKKPRPTRAEGSDVANAVLDGAD 357
SP|P14618-2|KPYM_HUMAN GIEIPAEKVFLAQKMMIGRCNRAGKPVICATQMLESMIKKPRPTRAEGSDVANAVLDGAD 357
SP|P14618-3|KPYM_HUMAN GIEIPAEKVFLAQKMMIGRCNRAGKPVICATQMLESMIKKPRPTRAEGSDVANAVLDGAD 342
TR|Q504U3|Q504U3_HUMAN GIEIPAEKVFLAQKMMIGRCNRAGKPVICATQMLESMIKKPRPTRAEGSDVANAVLDGAD 192
                       ************************************************************

SP|P14618|KPYM_HUMAN   CIMLSGETAKGDYPLEAVRMQHLIAREAEAAIYHLQLFEELRRLAPITSDPTEATAVGAV 417
SP|P14618-2|KPYM_HUMAN CIMLSGETAKGDYPLEAVRMQHLIAREAEAAMFHRKLFEELVRASSHSTDLMEAMAMGSV 417
SP|P14618-3|KPYM_HUMAN CIMLSGETAKGDYPLEAVRMQHLIAREAEAAIYHLQLFEELRRLAPITSDPTEATAVGAV 402
TR|Q504U3|Q504U3_HUMAN CIMLSGETAKGDYPLEAVRMQHLIAREAEAAMFHRKLFEELVRASSHSTDLMEAMAMGSV 252
                       *******************************::* :***** * :  ::*  ** *:*:*

SP|P14618|KPYM_HUMAN   EASFKCCSGAIIVLTKSGRSAHQVARYRPRAPIIAVTRNPQTARQAHLYRGIFPVLCKDP 477
SP|P14618-2|KPYM_HUMAN EASYKCLAAALIVLTESGRSAHQVARYRPRAPIIAVTRNPQTARQAHLYRGIFPVLCKDP 477
SP|P14618-3|KPYM_HUMAN EASFKCCSGAIIVLTKSGRSAHQVARYRPRAPIIAVTRNPQTARQAHLYRGIFPVLCKDP 462
TR|Q504U3|Q504U3_HUMAN EASYKCLAAALIVLTESGRSAHQVARYRPRAPIIAVTRNPQTARQAHLYRGIFPVLCKDP 312
                       ***:** :.*:****:********************************************

SP|P14618|KPYM_HUMAN   VQEAWAEDVDLRVNFAMNVGKARGFFKKGDVVIVLTGWRPGSGFTNTMRVVPVP 531
SP|P14618-2|KPYM_HUMAN VQEAWAEDVDLRVNFAMNVGKARGFFKKGDVVIVLTGWRPGSGFTNTMRVVPVP 531
SP|P14618-3|KPYM_HUMAN VQEAWAEDVDLRVNFAMNVGKARGFFKKGDVVIVLTGWRPGSGFTNTMRVVPVP 516
TR|Q504U3|Q504U3_HUMAN VQEAWAEDVDLRVNFAMNVGKARGFFKKGDVVIVLTGWRPGSGFTNTMRVVPVP 366
                       ******************************************************
```
