# Supplementary material for: PGCA: An algorithm to link protein groups created from MS/MS data
Source: PLoS One. 2017 May 31;12(5):e0177569. doi: 10.1371/journal.pone.0177569 (PMC5451011; doi:10.1371/journal.pone.0177569)
Supplement: S1 Appendix — (PDF) [file pone.0177569.s003.pdf]

APPENDIX for PGCA: an algorithm to link protein groups created from MS/MS data

David Kepplinger[1◑], Mandeep Takhar[2◑], Mayu Sasaki[2], Zsuzsanna Hollander[2], Derek Smith[3], Bruce McManus[2], W. Robert McMaster[4], Raymond T. Ng[2,5], Gabriela V. Cohen Freue[1*],

1 Department of Statistics, University of British Columbia, Vancouver, British Columbia, Canada
2 NCE CECR PROOF Centre of Excellence, Vancouver, British Columbia, Canada
3 University of Victoria - Genome BC Proteomics Centre, Victoria, British Columbia, Canada
4 Department of Medical Genetics, University of British Columbia, Vancouver, British Columbia, Canada
5 Department of Computer Science, University of British Columbia, Vancouver, British Columbia, Canada

◑These authors contributed equally to this work.
*** gcohen@stat.ubc.ca**

Proofs of properties

This Section presents proof outlines of the Lemma 1 and the Theorem 1 in the main text.

Lemma 1 *Let L be the set of local groups from a file F (i.e., L=CreateLocalGroups(F)), G be the set of global groups from a dictionary D, and $\mathcal{B} = L \cup G$. Let $\tilde{D} = UpdateDictionary(D, L)$ be the output dictionary from the UdpdateDictionary algorithm. Given B, a set of accession numbers from those in $\mathcal{B}$, and $\mathcal{A} = \{E \in \mathcal{B}$ such that $B \cap E = E\}$, B is a global group in $\tilde{D}$ if and only if $\mathcal{A}$ is non-empty and connected, and $B \cap E = \emptyset$, $\forall E \in \mathcal{B} \setminus \mathcal{A}$.*

Outline of Proof Lemma 1
$\Longrightarrow$) If B is a global group in $\tilde{D}$, by step 9 in Algorithm 3 in the main text, B resulted from updating a local group b in $\mathcal{B}$, and B can be written as the union of *all* sets in $\mathcal{B}$ connected with b (including b). Thus, by construction, these sets are included in B and form a non-empty and connected set $\mathcal{A}$. Moreover, if B overlaps a set C not in $\mathcal{A}$, then C is connected with b (by B), which is a contradiction.
$\Longleftarrow$) As $\mathcal{A}$ is non-empty and connected, then there exists a local group $b \in \mathcal{A}$, which, updated by UpdateDictionary, became a global group $\tilde{B}$ in $\tilde{D}$. It is sufficient to prove that $\tilde{B} = B$. By construction, $\tilde{B}$ can be written as the union of *all* sets in $\mathcal{B}$ connected with b (including b). As b is included B, any set E in $\mathcal{B}$ connected with b must also be connected with B. Moreover, as sets overlapping with B must be included in B, $E \in \mathcal{A}$, implying that $\tilde{B} \subseteq B$. Moreover, all sets in $\mathcal{A}$ are connected and thus connected with b. As B can be written as the union of all sets in $\mathcal{A}$, then $B \subseteq \tilde{B}$. This proves that $B = \tilde{B}$ is a global group in $\tilde{D}$.$\blacksquare$

Theorem 1 *Let L_i be the set of local groups from an input file F_i (i.e., L_i=CreateLocalGroups(F_i)) for $i = 1, \ldots, nFiles$, and $\mathcal{L}_{nFiles} = \bigcup_{i=1}^{nFiles} L_i$ be the universe of all these local groups. Starting from an empty dictionary D_0, let $D_{nFiles} = PGCA(D_0, F_1, \ldots, F_{nFiles})$ be the output dictionary from input files $F_i, i = 1, \ldots, nFiles$. Given B a set of accession numbers from those in $\mathcal{L}_{nFiles}$ and $\mathcal{A} = \{E \in \mathcal{L}_{nFiles} : \ B \cap E = E\}$, B is a global group in D_{nFiles} if and only if $\mathcal{A}$ is non-empty and connected, and $B \cap E = \emptyset$, $\forall E \in \mathcal{L}_{nFiles} \setminus \mathcal{A}$.*

Outline of Proof Theorem 1

To prove by induction, let $nFiles = 1$. By step 4 of Algorithm 1 in the main text, D_1=UpdateDictionary(D_0,L_1) and the result follows replacing L by L_1 and D by D_0 in Lemma 1. We now assume that the theorem is true for $nFiles = M$ and prove it for $nFiles = M + 1$.

$\Longrightarrow$) If B is a global group in D_{M+1}, $\mathcal{G}_M$ is the set global groups from D_M, and D_{M+1}=UpdateDictionary(D_M,L_{M+1}), by Lemma 1, B can be written as the union of all (connected) groups in $\mathcal{G}_M \cup L_{M+1}$ included in B. Any of these groups are either in $\mathcal{G}_M$, which by $nFiles = M$ can be written as the union of connected local groups from $\mathcal{L}_M$, or they are local groups in L_{M+1}. Thus, B can be written as the union of connected local groups from $\mathcal{L}_{M+1}$ included in B. Moreover, any local group H in $\mathcal{L}_{M+1}$ overlapping B is either a local group from L_{M+1}, thus, by Lemma 1 included in B, or H is a local group in $\mathcal{L}_M$. In the latter case, by $nFile = M$, it is easy to show that H is updated by PGCA-All into a global group in G_M, which by Lemma 1 is also included in B.■

$\Longleftarrow$) Suppose that B is not a global group in D_{M+1}. Then, by Lemma 1 with $\mathcal{B} = \mathcal{G}_M \cup L_{M+1}$, there either exists a set E in $\mathcal{G}_M \cup L_{M+1}$ such that $E \nsubseteq B$ and $B \cap E \neq \emptyset$, or there exist at least G_1 and G_2 in $\mathcal{G}_M \cup L_{M+1}$ such that $G_1 \subseteq B$ and $G_2 \subseteq B$ but G_1 and G_2 are not connected. By assumption, $E \in L_{M+1}$ results in a contradiction. Then, E is a global group in D_M. Using the results from $nFiles = M$, $E = \bigcup_{\{H \in \mathcal{A}\}} H$, where $\mathcal{A} = \{H \in \mathcal{L}_M,$ such that $E \cap H = H\}$ is non-empty and connected. $B \cap E \neq \emptyset,$ then $\exists H_0 \in \mathcal{A}$ such that $B \cap H_0 = H_0$. As all H in $\mathcal{A}$ are connected, then $B \cap H = H$ for all $H \in \mathcal{A}$. Then, $B \cap E = \bigcup_{\{H \in \mathcal{A}\}} B \cap H = E$, which is a contradiction. Thus, there exist at least G_1 and G_2 in $(\mathcal{G}_M \cup L_{M+1})$ both included in B and not connected. Using the results of $nFiles = M$, both sets can be written as the union of local sets from $\mathcal{L}_M$ and local sets from L_{M+1}. Thus, there exists $H_1 \in L_j$, for some $j \leq M + 1$, and $H_2 \in L_s$, for some $s \leq M + 1$, such that $H_1 \subseteq G_1 \subseteq B$, $H_2 \subseteq G_2 \subseteq B$. Then, both H_1 and H_2 are in $\mathcal{A}$ and thus are connected, which is a contradiction as the connector set would also connect G_1 and G_2. Thus, B is a global group in D_{M+1}.■
